# Pancreas-preserving segmental duodenectomy for duodenal malignancy: two case reports

**DOI:** 10.3389/fonc.2026.1872839

**Published:** 2026-08-05

**Authors:** Junpeng Wang, Longchang Chen, Yan Sun, Zheng Wang, Ying Guo, Liuxin Duan, Quanda Liu

**Affiliations:** Department of General Surgery, Guang’anmen Hospital, China Academy of Chinese Medical Sciences, Beijing, China

**Keywords:** case repors, duodenojejunostomy, pancreas-preserving duodenectomy, secondary duodenal malignancy, segmental duodenectomy

## Abstract

The extent of resection required for duodenal malignancy has long been debatable. Compared with pancreaticoduodenectomy (PD), pancreas-preserving segmental duodenectomy (PPSD) is a less demolitive procedure that provides definitive treatment for a range of duodenal pathologies, including adenocarcinoma. However, the rarity of these malignancies limits large-scale clinical studies on the feasibility and safety of PPSD. Thus, case reports and small case series may provide some clinical guidance for this rare situation. We herein report two cases of patients with duodenal malignancy who were treated with PPSD. Case 1 was a 36-year-old female patient, who was diagnosed with adenocarcinoma of the hepatic flexure with synchronous invasion of the descending portion of the duodenum. After 4 cycles of neoadjuvant chemotherapy, she received radical right colectomy and PPSD. Case 2 was a 45-year-old male patient, who was found to have a metachronous metastasis in the horizontal portion of the duodenum 14 months after laparoscopic extended right colectomy with complete mesocolic excision for transverse colon adenocarcinoma. The duodenal lesion was confirmed by imaging, endoscopic, and pathological examinations. He received PPSD combined with a partial resection of the uncinate process of the pancreas. Postoperative pathology confirmed R0 resection in both cases, and both patients had an uneventful recovery. These cases provide further evidence supporting the feasibility and safety of PPSD for non-ampullary duodenal tumors. However, the long-term oncological outcomes are still unclear; therefore, further studies with longer follow-up and larger sample sizes are warranted.

## Introduction

Duodenal adenocarcinoma is a rare malignancy, accounting for less than 1% of all gastrointestinal cancers (1, 2). Pancreaticoduodenectomy (PD) remains the standard surgical procedure for duodenal malignancy due to common anatomy, blood supply, nodal retrieval, and margin acquisition (3). However, PD may be too aggressive for localized and non-ampullary duodenal malignancy. PPSD is an attractive option, as it shortens the operative time, avoids resection of the proximal pancreas and distal bile duct, and eliminates the need for biliary and pancreatic anastomoses required in PD (4). Some studies comparing PPSD and PD have shown no difference in overall or disease-specific survival, despite a lower lymph node yield with PPSD (5–8). We herein report two cases of duodenal adenocarcinoma treated with PPSD. The short-term clinical outcomes and safety are encouraging.

## Case 1

**Figure 1:**
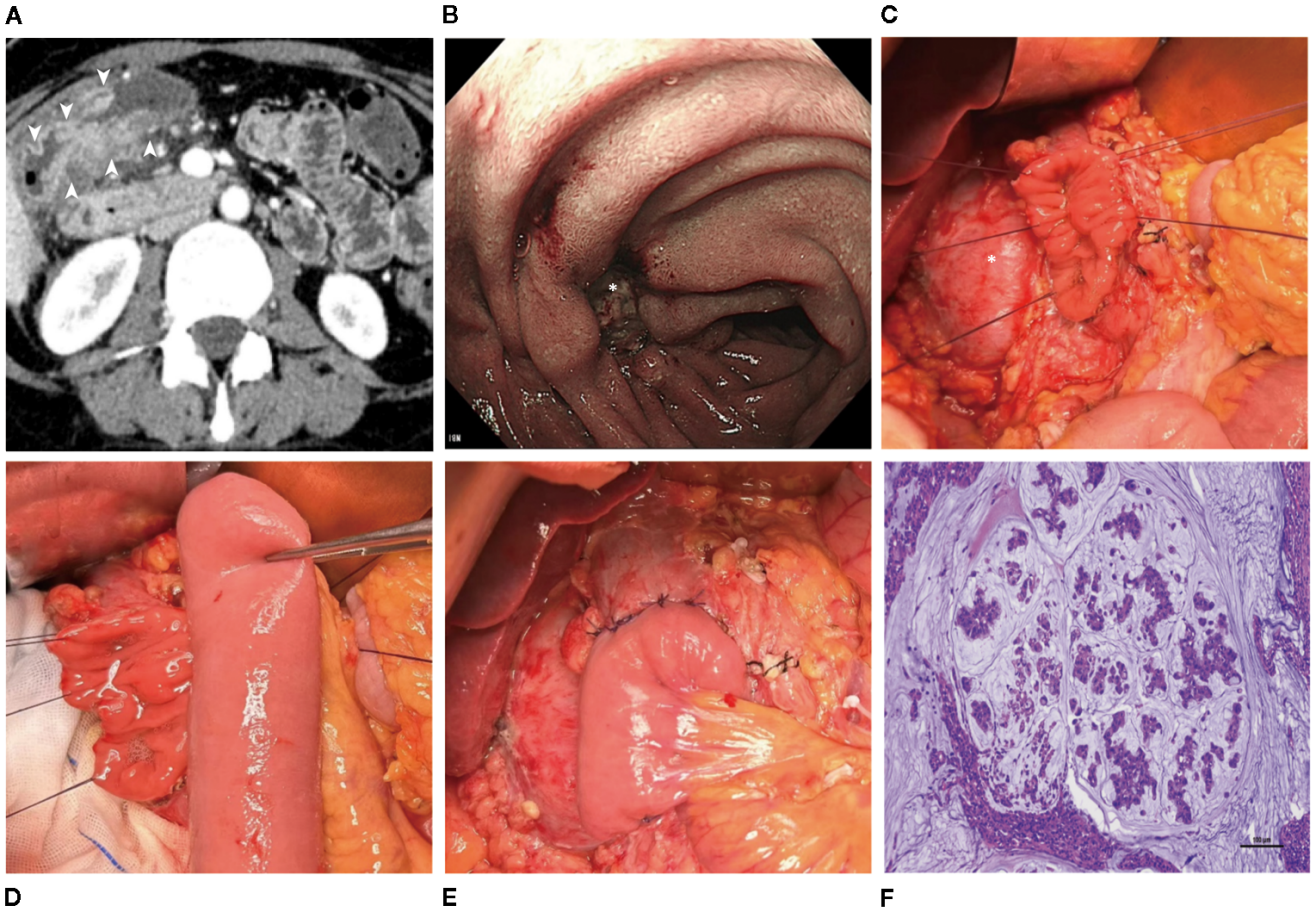
Preoperative imaging, intraoperative photographs, and pathology of case 1. Computerized tomography image showed an extensive hepatic flexure colon malignancy (arrow) with an invasion of the duodenum **(A)**. Gastroduodenoscopy revealed an irregularly ulcerous duodenal lesion [**(B)**, asterisk]. The intraoperative pictures about pancreas-preserving segmental duodenectomy: after en bloc removal of colon and duodenal malignancy and adjacent tissues, showing the lateral incision of the descending duodenum and the right kidney [**(C)**, asterisk], performing a duodenojejunal Roux-en-Y anastomosis **(D)**, and completion of the digestive tract reconstruction **(E)**. Pathology of the duodenal surgical specimen confirmed mucinous adenocarcinoma **(F)**.

**Figure 2:**
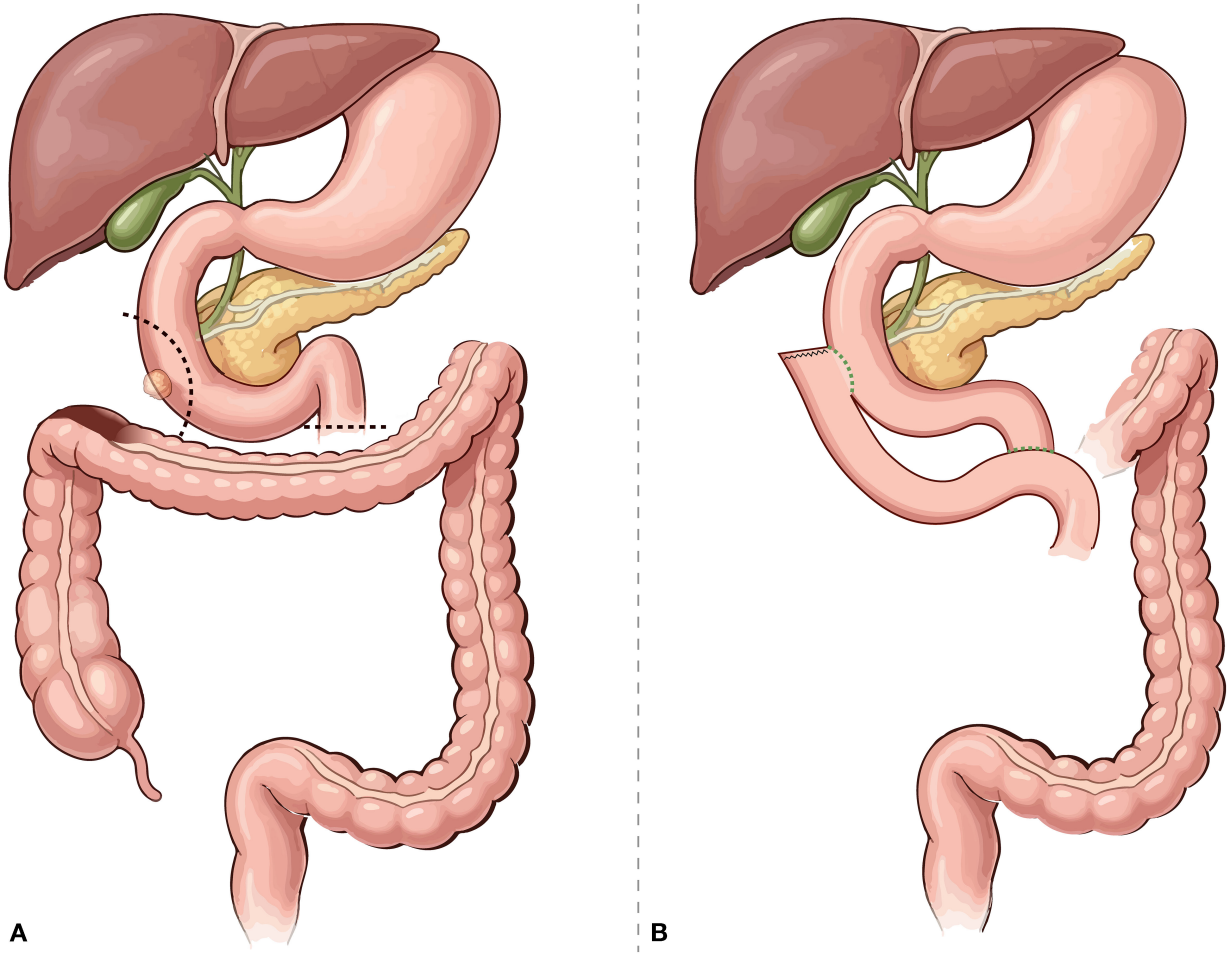
Pancreas-preserving segmental duodenectomy for malignancy in the descending portion of the duodenum in case 1. The lateral wall of the duodenum was excised by wedge resection, with clear 2-cm negative margins, and en bloc removal with the right hemicolectomy specimen **(A)**. A duodenojejunal Roux-en-Y anastomosis was constructed **(B)**.

In February 2023, a 36-year-old woman presented with abdominal pain. Physical examination and laboratory tests, including tumor markers, revealed no significant abnormalities. Abdominal contrast-enhanced computerized tomography (CT) scan demonstrated a mass at the hepatic flexure of the colon and subtle wall thickening of the descending portion of the duodenum. Colonoscopic biopsy confirmed the diagnosis of adenocarcinoma. Subsequently, she received neoadjuvant chemotherapy consisting of 4 cycles of oxaliplatin and capecitabine (XELOX). Upon completion of neoadjuvant chemotherapy, the patient was referred to our department for further evaluation. Abdominal contrast-enhanced CT demonstrated an extensive tumor at the hepatic flexure of the colon, with irregular wall thickening measuring 8 cm in length and an invasion of the adjacent duodenum (**Figure 1A**). Colonoscopic biopsy revealed colonic mucinous adenocarcinoma. Gastroduodenoscopy showed an ulcerative lesion in the descending duodenum (**Figure 1B**). In August 2023, radical right hemicolectomy combined with wedge resection of the descending portion of the duodenum was performed (**Figure 1C**). Intraoperatively, the Valdoni Cattell Braasch maneuver was used to adequately expose the pancreatic head, duodenum, and retroperitoneal structures. The lesion was situated on the opposite side of the ampulla of Vater. Given the adequate distance from the ampulla, the duodenal wall was directly incised along the lesion and the ampulla of Vater was carefully identified and protected. The proximal jejunum was transected, and a duodenojejunal Roux-en-Y anastomosis was constructed to restore alimentary tract continuity (**Figures 1D, E**). The schematic diagram of the surgical procedures is shown in **Figure 2**. Final pathology of the duodenal specimen confirmed mucinous adenocarcinoma (**Figure 1F**). A total of 29 lymph nodes (LNs) (3 periduodenal) were retrieved from the surgical specimen; no metastatic LNs were identified (pT4bN0M0). Her postoperative course was uneventful, and she experienced no complications. She soon received six cycles of adjuvant leucovorin, fluorouracil, and oxaliplatin (FOLFOX) chemotherapy. At the postoperative 3-year follow-up, she remains disease-free.

## Case 2

**Figure 3:**
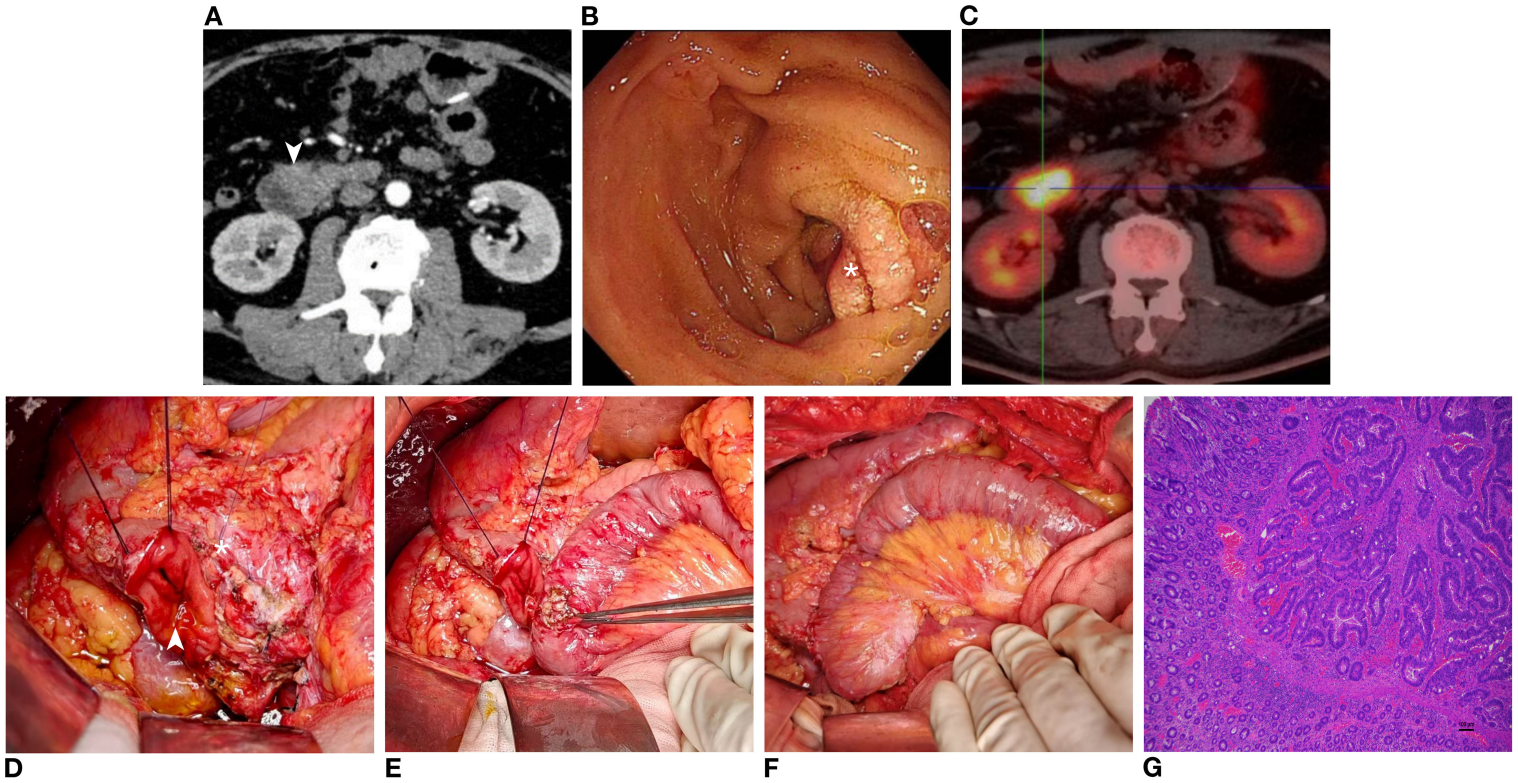
Preoperative imaging, intraoperative photographs, and pathology of case 2. Computerized tomography image showed a malignant lesion of a size of 27 mm × 33 mm × 23 mm in the horizontal portion of the duodenum [**(A)**, arrow]. Gastroduodenoscopy revealed an irregularly ulcerous duodenal lesion [**(B)**, asterisk]. PET-CT demonstrated a high fluorodeoxyglucose uptake and hypermetabolic lesion in the horizontal part of the duodenum **(C)**. The intraoperative pictures about pancreas-preserving segmental duodenectomy: after en bloc removal of duodenal malignancy and adjacent tissues, showing the duodenal stump sparing the ampulla of Vater (arrow) and the head of the pancreas [**(D)**, asterisk], performing an end-to-side duodeno-jejunal anastomosis **(E)**, and completion of the digestive tract reconstruction **(F)**. Pathology of the duodenal specimen confirmed a moderately to poorly differentiated adenocarcinoma **(G)**.

**Figure 4:**
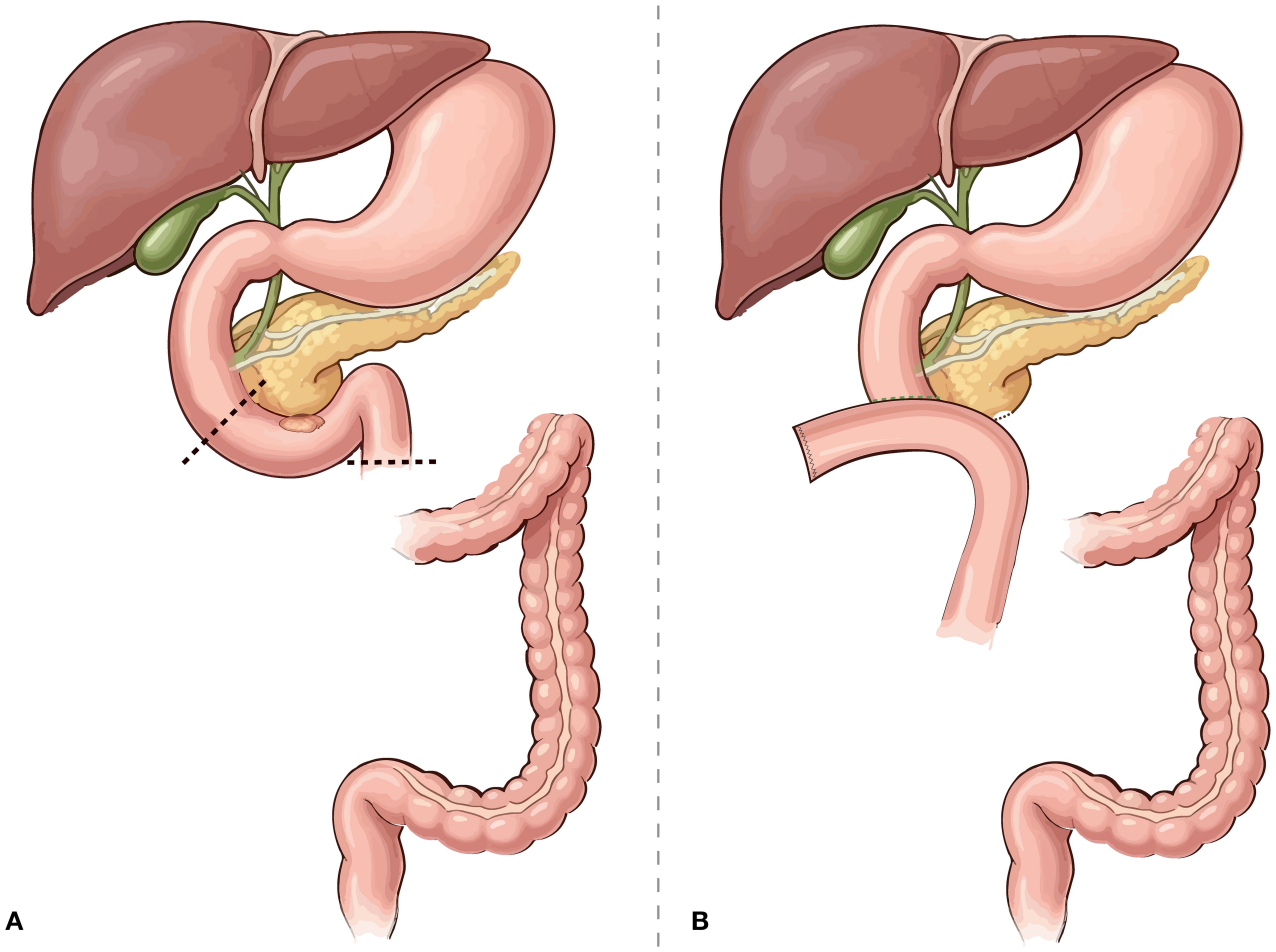
Pancreas-preserving segmental duodenectomy for malignancy in the horizontal portion of the duodenum in case 2. The horizontal portion of the duodenum was transected with a 2-cm margin lateral to the right aspect of the tumor, together with partial resection of the pancreatic uncinate process **(A)**. An end-to-side duodenojejunal anastomosis was then constructed **(B)**.

In February 2024, a 45-year-old man was referred to our department with transverse colon adenocarcinoma. He subsequently received laparoscopic extended right hemicolectomy with complete mesocolic excision. A paracolic tumor nodule and 38 LNs were retrieved; no metastatic LNs were identified (pT4aN1cM0). Subsequently, he received 8 cycles of adjuvant XELOX chemotherapy. In March 2025, imaging and histopathological evaluation revealed a duodenal adenocarcinoma in the right aspect of the horizontal portion of the duodenum, consistent with metachronous metastasis from the previously treated colon primary. He was asymptomatic, with no abnormal physical findings or laboratory test results other than an elevated serum CA19–9 level of 73.1 IU/mL (reference range, 0–37 IU/mL). Abdominal CT demonstrated a 27 mm × 33 mm × 23 mm lesion in the right aspect of the horizontal portion of the duodenum (**Figure 3A**). Gastroduodenoscopy identified an irregular ulcerated lesion (**Figure 3B**). Histopathological examination confirmed moderately differentiated adenocarcinoma. Microsatellite instability testing was performed on the tumour, together with immunohistochemistry for mismatch repair protein expression; the results did not suggest Lynch syndrome. PET-CT demonstrated increased fluorodeoxyglucose uptake (SUVmax = 13.4) and a hypermetabolic lesion in the horizontal portion of the duodenum (**Figure 3C**). The duodenal adenocarcinoma was adjacent to the uncinate process of the pancreas. The patient declined the surgical option of an extended Whipple procedure and had a strong demand for preservation of the pancreas. PPSD was then performed. The procedure involved segmental duodenectomy with proximal jejunal resection, partial resection of the pancreatic uncinate process, and LN dissection. Finally, an end-to-side duodenojejunal anastomosis was constructed (**Figures 3D–F**). The schematic of the surgical procedure is shown in **Figure 4**. Final pathology of the duodenal specimen confirmed a moderately to poorly differentiated adenocarcinoma (**Figure 3G**). A total of 5 periduodenal LNs were retrieved from the surgical specimen; none were positive for metastasis. The resection margins of small intestine and the uncinate process of the pancreas were microscopically negative. Postoperative drain fluid amylase levels were routinely monitored, and no pancreatic fistula, chylous fistula, or other severe complications occurred. The patient subsequently received 6 cycles of adjuvant FOLFOX chemotherapy. Serum CA 19-9 decreased to 24.7 IU/mL one week postoperatively. During the follow-up period, serum CA 19-9 levels fluctuated between 24.9 and 32.0 IU/mL.

## Discussions

Although both patients had colon cancer involving the duodenum, the cases represented distinct patterns of disease. Case 1 involved direct duodenal invasion by a hepatic-flexure adenocarcinoma, an uncommon presentation (9). This rarity may reflect earlier diagnosis of right-sided colon cancers before local extension to the duodenum occurs. In this setting, the surgical objectives are complete oncologic resection and prevention of impending complications, including obstruction and enteric fistula. Curative treatment requires en bloc resection of all involved tissue with right colectomy. However, multiorgan resection for colorectal carcinoma is often associated with higher postoperative morbidity and mortality. When duodenal involvement is limited and the ampulla of Vater is spared, PPSD may be an appropriate alternative if negative margins can be achieved. In addition, colon adherence to the duodenum does not invariably signify malignant invasion. In some patients, it represents inflammatory change alone, in which case limited duodenal resection and closure may be sufficient (10). Because preoperative imaging cannot always distinguish tumor infiltration from inflammatory adhesion, definitive assessment may depend on intraoperative findings (11). In appropriately selected patients, radical right colectomy combined with PPSD may provide adequate oncologic control without excess operative risk.

By contrast, case 2 involved metachronous duodenal metastasis from transverse colon cancer, which is also rare (12, 13). In the absence of other distant disease, this lesion was considered isolated. Although the mechanism of spread remains uncertain, lymphatic spread through periduodenal nodes has been proposed (14). For patients with a prior curative colorectal resection and no evidence of local recurrence, complete resection of an isolated duodenal metastasis is warranted; neoadjuvant therapy may also be considered in selected cases. More broadly, this case highlights the biologic plausibility of delayed metastatic recurrence, potentially related to early tumor cell dissemination and subsequent dormancy (15). Accordingly, surveillance after curative intent treatment remains essential to identify recurrence or metastasis at a potentially treatable stage.

PD has been the standard surgical procedure for managing duodenal malignant tumors; however, it is associated with a complication rate of 30%-60%, including delayed gastric emptying, pancreatic fistula, bile leak, and surgical site infection (16). PPSD obviates pancreaticoenteric anastomosis, thereby markedly reducing the morbidity associated with PD. The optimal surgical procedure for duodenal malignancy has long been debated (17). PD has been advocated for all patients with duodenal malignancy, regardless of location, so as to ensure adequate wide margin clearance and regional lymphadenectomy (18). In contrast, PPSD has been proposed for non-ampullary duodenal malignancy, provided a margin-negative resection can be achieved (7, 19, 20). Rather than uniformly advocating PD, the surgical strategy should balance perioperative risk against oncologic radicality. Although Cirocchi et al. (21) reported higher 5-year overall survival with PD for duodenal invasion by locally advanced right colon cancer, they also found substantially greater 30-day morbidity after PD than after PPSD (12.8% vs. 0%), largely related to pancreaticojejunal anastomotic complications. Mitchell et al (22). and Chung et al (23). similarly described PPSD as safe and effective in selected patients, with lower morbidity and shorter hospital stays. A consequent question is whether this reduction in complications compromises survival because of inadequate oncologic resection or recurrence. Patel et al. (24) found that PPSD achieved margin-negative resection and LN sampling comparable to PD, and Busquets et al. (25) reported acceptable postoperative outcomes, with severe complications in 14% and postoperative mortality in 3%, without observed recurrence during follow-up. In their study of distal duodenal tumors, Onkendi et al. (8) found that survival was improved by curative resection without being compromised by limited resection, while also noting no significant association between extent of resection and survival; rather, pathologic grade and advanced tumor stage were associated with worse prognosis. Platoff et al. (17) reported no survival disadvantage with PPSD compared with PD and suggested that adjuvant therapy may have a greater influence on survival than surgical approach. These studies support PPSD as a reasonable alternative to PD in selected patients, although this interpretation remains limited by the retrospective design and potential selection bias of the available literature. **Table 1** summarizes relevant studies supporting PPSD for duodenal tumors.

**Table 1:** Summary of reported cases treated with pancreas-preserving segmental duodenectomy.

Author	Year	No. of cases	Histopathology	Anastomosis	Median length of stay (days)	Complications	Outcomes
Onkendi et al. (8)	2012	28	28 adenocarcinomas	NR	NR	4 anastomotic leaks 2 DGE	The 5-yr and 10-yr survival rates were 52% and 43%, respectively.
Stauffer et al. (35)	2013	10	2 adenocarcinomas 5 adenomas 3 others	7 side-to-side DJ 2 end-to-side DJ 1 GJ	5.6	1 PF with DGE 1 episode of medical deconditioning	No recurrence was observed at a median follow-up of 51 mos.
García et al. (26)	2015	8	1 adenocarcinoma 5 GISTs 2 others	1 end-to-side DJ 6 end-to-end DJ 1 primary closure	9.7	1 readmission 1 grade B PF with DGE 1 multiple organ dysfunction syndrome	Adenocarcinoma: Liver metastasis at 2 mos; death at 12 mos.
Cloyd et al. (7)	2015	746	746 adenocarcinomas	NR	NR	Thirty-days and 90-days postoperative mortality rates were 9.0% and 13.0%, respectively.	The median disease-specific survival and OS were 75 mos and 38 mos, respectively.
Dorcaratto et al. (19)	2015	11	4 adenocarcinomas 5 adenomas 1 GIST 1 other	5 end-to-side DJ 5 end-to-end DJ 1 side-to-side DJ	14	1 bleeding episode 1 anastomotic leak	Adenocarcinoma: One patient died at 7 mos. The mOS and DFS were 13 mos and 13 mos, respectively.
Mitchell et al. (22)	2017	19	6 adenocarcinomas 5 adenomas 4 GISTs 4 others	19 side-to-side DJ	8	1 recurrent adenocarcinoma 1 anastomotic stricture 1 incisional hernia	Adenocarcinoma: One patient died at 18 mos. Alive and well: 1 patient < 1 yr, 2 patients > 1 yr, 1 patient > 6 yrs, 1 patient > 12 yrs.
Blanco et al. (34)	2020	12	6 adenocarcinomas 4 GISTs 2 others	11 side-to-side DJ 1 end-to-end DJ	14	2 digestive hemorrhage episodes 1 anastomotic dehiscence 2 bleeding episodes 1 DGE	Adenocarcinoma: The 1-, 3-, and 5-yr OS rates were 100%, 50%, and 25%, respectively; The 1-, 3-, and 5-yr DFS rates were 83%, 50%, and 33%, respectively; The mOS and DFS were 47.1 mos and 43.1 mos, respectively.
Busquets et al. (25)	2021	35	4 adenocarcinomas 1 adenoma 13 GISTs 17 others	27 end-to-end DJ 1 ampullary-jejunal suture 7 primary closures	8-14	5 abscesses 1 grade A and 2 grade B PF 3 DGE 1 anastomotic dehiscence 1 episode of hemorrhage 1 pneumonia	Adenocarcinoma: One patient died at 1 mo. Alive and well: 1 patient < 1 yr, 1 patient > 5 yrs, 1 patient > 7 yrs.
Platoff et al. (17)	2021	878	878 adenocarcinomas	NR	NR	NR	The mOS was 46.7 mos.
Patel et al. (24)	2023	31	14 adenocarcinomas 5 adenomas 5 GISTs 6 others	9 GJ 21 side-to-side DJ 1 end-to-side DJ	9	4 abscesses 1 arterial bleeding episode 1 grade B PF 1 duodenal fistula	Adenocarcinoma: Four patients died at 2, 4, 12.2, and 34 mos, respectively. Alive and well: 2 patients < 1 yr, 3 patients ≥ 2 yrs, 1 patient > 3 yrs, 1 patient > 4 yrs, 2 patients > 5 yrs, 1 patient > 11 yrs.
Chung et al. (23)	2024	29	8 adenocarcinomas 11 adenomas 7 GISTs 3 others	1 end-to-end GJ 5 Heineke-Mikulicz repairs 23 side-to-side DJ	6	2 DGE 2 reoperations 5 readmissions	Adenocarcinoma: Three patients died at 12 days, 5 ms, and 5 mos, respectively. Alive and well: 1 patient > 4 yrs, 1 patient > 5 yrs, 1 patient > 6 yrs, 2 patients > 9 yrs.

*GIST* gastrointestinal stroma tumor, *NR* not reported, *DJ* duodenojejunostomy, *GJ* gastrojejunostomy, *PF* pancreatic fistula,.

*DGE* delayed gastric emptying, *OS* overall survival, *DFS* disease-free survival

Regional LN status has uniformly been shown to have a considerable impact on long-term survival after curative resection of primary duodenal malignancy (26). Resection with curative intent, either PD or PPSD, and the absence of LNs metastasis favors survival in patients with duodenal malignancy (6, 17). LNs dissection is generally recommended for tumors extending beyond the submucosa, although it may be omitted for intramucosal lesions (27). Previous studies have demonstrated that lymphadenectomy at the time of surgery is associated with improved survival (28). However, the optimal number of retrieved LNs is not well established. The AJCC guidelines recommend the examination of at least six LNs for accurate N staging of duodenal or small bowel adenocarcinoma. However, some have questioned whether this minimal number of LNs should be examined (29). Sarela et al. (30) reported that examination of ≥15 regional LNs improved prognostic stratification by pN category. The National Comprehensive Cancer Network Clinical Practice Guidelines in Oncology recommend that the goal for all small bowel adenocarcinoma resections be the retrieval of at least 8 regional LNs for evaluation (5).

Although most prognostic data are derived from primary tumors, nodal status remains relevant in metastatic duodenal malignancies. Zhou et al. (31) reported a case of metachronous duodenal metastatic melanoma with regional nodal involvement identified intraoperatively, supporting consideration of regional lymphadenectomy even in selected metastatic lesions. Tumors arising in or extending to the duodenum often warrant dissection of nodal basins around the superior mesenteric vessels (23, 25, 32). According to the Union for International Cancer Control TNM classification, regional LNs for nonampullary duodenal tumors include the pancreaticoduodenal, pyloric, hepatic, and superior mesenteric nodes. Because nodal spread follows predictable lymphatic pathways, removal of upstream nodal stations may improve oncologic outcomes (33). On this basis, superior mesenteric nodes were dissected in Case 1 (3 LNs retrieved), whereas both peripancreatic head and superior mesenteric nodal stations were dissected in Case 2 (5 LNs retrieved).

PPSD may not be suitable for all patients with duodenal malignancy. In carefully selected patients with tumors that do not involve the ampulla of Vater or pancreas, the technique can provide an appropriate resection. In Case 1, PPSD was considered more appropriate because the duodenal lesion was relatively localized, with invasion of the duodenal wall on the side opposite the ampulla of Vater. This configuration enabled complete (R0) resection of the duodenal invasion while preserving the pancreas. In Case 2, the patient declined an extended PD and wished to preserve the pancreas. Although the patient achieved curative resection, we still consider that he is not an ideal candidate for PPSD because his duodenal tumor lay in close proximity to the pancreas. The distance between the lesion and the resection margin was close, representing a risk factor for local recurrence or distant metastasis. Therefore, the choice of PPSD may have compromised the radicality of resection.

Reconstruction after resection is typically accomplished with end-to-end, end-to-side, or side-to-side duodenojejunostomy, or via a Roux-en-Y configuration (19). Existing evidence indicates that there were no significant differences in morbidity rates or in postoperative length of stay between those who had end-to-end or end-to-side anastomoses (19). The duodenojejunal anastomosis can also be performed as a side-to-side one and has been performed as laparoscopic surgery (22). High-quality evidence comparing digestive tract anastomotic techniques remains limited; thus, no definitive conclusion can be drawn regarding the optimal approach. In practice, the selection of anastomotic technique is largely guided by the surgeon’s experience, the anatomical location of the tumors, and anastomotic tension.

In conclusion, we agree that in tumors confined to the duodenum, PD may be overly aggressive surgery, and segmental or wedge resection, would be suitable for these tumors, if the ampulla of Vater and the head of the pancreas are not involved and radical resection margins can be accomplished. Dissection of LN stations situated upstream in the lymphatic flow may be beneficial. Our findings provide evidence supporting the safety and feasibility of PPSD for carefully selected patients with duodenal malignancy. However, the long-term oncological outcomes are still unclear; therefore, further studies with longer follow-up and larger sample sizes are warranted.

## Data Availability

The original contributions presented in the study are included in the article/supplementary material. Further inquiries can be directed to the corresponding author.
